# Guild diversity impacts demographic outcomes of novel species interactions following range shifts

**DOI:** 10.1111/1365-2656.70108

**Published:** 2025-07-25

**Authors:** Michael O'Connor, Lesley T. Lancaster

**Affiliations:** ^1^ School of Biological Sciences University of Aberdeen Aberdeen UK

**Keywords:** biotic resistance, community diversity, competition, complex life cycles, novel interactions, range shifting

## Abstract

1. Novel competitive interactions between native and range shifting species can precipitate local extinction of native species. However, increased biological complexity within recipient communities may prevent native species loss by decreasing the strength of novel competition experienced by any one species. This phenomenon, termed ‘biotic resistance’, is commonly applied in invasion ecology, but has received little attention in the context of climate induced range shifts.
2. Here we investigate the effects of biotic resistance in competition between resident native and range‐shifting damselflies in a region of Scotland newly colonised by the range‐shifter, using competitive mesocosm treatments across multiple life stages and experimental temperatures.
3. Our focal native species (*Lestes sponsa*) was unaffected by increasing competitive complexity as larvae, showing no fitness benefits in multispecies treatments compared to intraspecific or even interspecific scenarios in the presence of the range shifter. However, multispecies competition with both native and range‐shifting species improved adult survival of our focal native species at higher temperatures, compared to interspecific competition with just the range shifter. For our focal range‐shifting species (*Ischnura elegans*), larval growth rate was significantly reduced in multispecies treatments compared to intraspecific or two‐species interspecific scenarios, yet adult range shifter survival showed no decrease in multispecies treatments. Furthermore, range shifter larvae displayed improved survival and growth in colder temperatures, compared to a lack of any temperature effects on adult survival.
4. These results suggest that biotic resistance may alleviate the competitive impacts of range shifters on native communities by providing a life stage‐dependent benefit to native species while simultaneously decreasing the fitness of range shifters. However, shifting temperatures can cause this interaction to swap between competition and facilitation, creating an environmentally dependent scenario that may benefit both range shifters and resident species, promoting the maintenance of diversity in high latitude communities.

## Graphical abstract

This study applies the theory of biotic resistance, a common concept in invasion ecology, to the world of climate driven range shifts, and finds support that biotic resistance may create a life‐stage dependent benefit to resident species, alleviating the competitive impacts of range shifters on native communities.

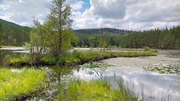

## 1 INTRODUCTION

Range shifting is a common response to climate change, whereby environmental warming results in changes to individual survival and dispersal rates, driving shifts in overall species distributions (Thomas, 2010; Walther et al., 2002). Such shifting may allow species to mitigate adverse impacts of global warming (Parmesan & Yohe, 2003), and offers the possibility of range expansion into new regions that were previously below a species' minimum thermal limits (Hickling et al., 2005; Lancaster et al., 2015). This kind of climate tracking can thus be beneficial to a species' survival, and as such range shifter dynamics are a well‐studied aspect of movement ecology and climate change research (Lawlor et al., 2024). However, novel competitive interactions between range shifters and the species native to the habitats they are entering have the potential to lead to species loss (Pauchard et al., 2016; Sorte et al., 2010; Urban et al., 2012), particularly of native species that cannot compete with the arrival of a strong novel competitor (Alexander et al., 2015; Gilman et al., 2010). As examples of such native species loss in response to range shifter competition become more widespread (Adams et al., 2023; Brooker et al., 2007; Fitt & Lancaster, 2017), the importance of investigating the competitive impact of climate‐mediated range shifts on resident communities becomes more apparent.

One area we can look to for insight into the potential mechanisms and impacts of range shifter competition is that of invasive species ecology, given the parallels in novel competitive dynamics between invasives and range‐shifting species (Wallingford et al., 2020). An important, hypothesised factor in determining the success of non‐native invasive species, and so potentially too of range shifters, is that of biotic resistance (Beaury et al., 2020), which posits that the greater the biodiversity of a receiving community, the more likely it is to resist invasion from a nonnative species, due to the increased competitive environment already present in the community (Levine et al., 2004). For the nonnative species, this can translate into anything from reduced fitness to complete exclusion from the novel habitat (Gu et al., 2014). Conversely, from the view of the native species, greater biodiversity should therefore restrict the impacts of novel competition felt by any one species, reducing negative demographic impacts and ultimately the likelihood of resident species loss. We would expect, then, that lower‐diversity communities (such as those typically found at higher latitudes; Mittelbach et al., 2007), lacking this competitive buffer, would thus be more likely to experience loss of native species when faced with a nonnative invader or range shifter (Beshai et al., 2023). Additionally, the ability of community diversity to dampen the competitive impacts of a nonnative species may be greater in low‐resource environments, due to stronger competition for a more limited habitat space (Stachowicz & Byrnes, 2006). This makes the concept of biotic resistance all the more important to range shifter dynamics, given that climate‐mediated range shifting typically occurs upslope or to higher latitudes (Guo et al., 2012), into more resource‐restricted, environmentally harsher habitats (Currie et al., 2004; Parmesan & Yohe, 2003).

While temperature changes form the driving context behind range shifting, they also play an important role in regulating the outcome of novel competitive interactions. The temperature dependency of competition is well‐established (Accolla & Forbes, 2021; Amarasekare & Coutinho, 2014; Hu et al., 2023; Olsen et al., 2016), and it plays an important role in determining species boundaries in high latitude environments (Anderegg & HilleRisLambers, 2019), interacting with biotic interactions to either restrict or facilitate range expansion (Sirén & Morelli, 2020). However, competition between range shifters and native species is expected to favour the range shifters, which tend to exhibit broader niche tolerances as an evolutionary consequence of their recent range expansions across heterogeneous habitats and environmental gradients (Lancaster, 2022). In contrast, native species are experiencing the dual pressures of novel competition from a range shifter as well as increased warming within their own ancestral distributions (Lord & Whitlatch, 2015). The outcome of competition between range shifting and native populations at range boundaries is thus expected to be highly temperature dependent (Alexander et al., 2015; Lancaster et al., 2017), with warmer conditions expected to provide a compounded competitive advantage to the range shifter, especially in areas where native communities are species‐poor (as discussed above).

The impact of these interacting mechanisms on competitive outcomes may be further complicated in species that exhibit complex life histories (Gómez‐Llano et al., 2023). This is especially true for those which undergo dramatic ontogenetic shifts in habitat between juvenile and adult stages (Moll & Brown, 2008; Werner, 1988), as this can often lead to differing competitive strategies between life stages (Bissattini et al., 2019). Such profound, within‐individual shifts in habitat and competitive strategy can alter species coexistence dynamics, as the mechanisms that dictate competition and coexistence in one life stage will notnecessarily translate over to the next; or if they do, can have alternative effects, swapping from facilitation to exclusion or vice versa (Moll & Brown, 2008). Moreover, individual life history functions specific to either adults (e.g. reproduction) or juveniles (e.g. growth) may vary in sensitivity to environmental conditions and competition. As such, understanding how competition and environments act and interact across life stages is vital to revealing the true outcomes of novel competition between range shifting and resident species.

Damselflies (Odonata: Zygoptera) are the ideal system in which to study these mechanisms of novel competition. Their complex life cycles are split between aquatic larval and terrestrial adult stages; larvae compete primarily for food while territorial adults compete for both mates and habitat/oviposition space, creating variation in both the context and outcomes of interactions within their respective environments. Importantly, many damselflies are undergoing dramatic contemporary range shifts, expanding into higher latitudes and altitudes in response to anthropogenic warming (Hickling et al., 2005). In northern Europe, the blue‐tailed damselfly *Ischnura elegans* is rapidly expanding its northern range limits (Hickling et al., 2005; Lancaster et al., 2015; Sánchez‐Guillén et al., 2016), and the novel competitive pressure it generates is believed to be suppressing populations of native, high‐latitude damselfly species, in particular the emerald damselfly, *Lestes sponsa* (Fitt & Lancaster, 2017). However, evidence for this suppression is based on correlative modelling of population densities, as well as laboratory interactions focusing solely on adult damselfly temperature preferences, and does not take into account either the species' complex life histories or wider community interactions.

In this study we investigated the outcomes of novel competitive interactions between range shifting and native species, with particular focus on native species demographic outcomes, and with specific regard to the impacts of community biodiversity (biotic resistance) and temperature and how their effects may interact across a species' complex life history. To achieve this, controlled competition experiments were carried out between range shifting (*Ischnura elegans*) and native (*Lestes sponsa*) damselfly species in both laboratory and semi‐wild mesocosm conditions for both larval and adult life stages. Individuals used in the experiment were captured near the northern (expanding) border of *I*. *elegans'* range in Scotland, in the same locations where Fitt and Lancaster et al. (2017) suggest *L*. *sponsa* populations suffer from the competitive impacts of this range shift. For these competition experiments, intraspecific (one species), interspecific (two species) and more complex multispecific (three species) treatments were used to test the impacts of increasing native community diversity on the outcomes of competition between the range shifter *I*. *elegans* and two native species (the focal native species *L*. *sponsa* and an additional native species ubiquitous at high latitudes, *Enallagma cyathigerum*; Brooks, 2018). Despite the closer phylogenetic relationship between *I*. *elegans* and *E. cyathigerum*, we opted to use *L*. *sponsa* as our focal native species due to its recorded history of competitive interactions with *I*. *elegans* (Fitt & Lancaster, 2017). Larval experiments were carried out at both ambient (15°C) and high (20°C) temperatures to test for potential interactive effects of competition and warming on native species demographics. These temperatures also reflect the current and predicted summer water temperatures of real‐world high latitude damselfly habitats, where the effects of biotic resistance are predicted to be most apparent (Stachowicz & Byrnes, 2006). Demographic parameters for larval growth rate, adult and larval survival, and adult female mating harassment under temperature‐dependent competition were recorded for the focal native *Lestes sponsa*, while larval growth, larval survival and adult survival parameters were also recorded for the range shifting *Ischnura elegans*.

We predicted that, in line with current biotic resistance theory, increased community biodiversity would reduce the demographic success of the range shifter due to the stronger competitive environment, while increasing the success of native species by diluting the impact of the novel competitive range shifter over a greater number of species. However, we also predicted a potential interaction between increasing diversity and temperature, as higher temperatures would preferentially support the range shifter due to its recent arrival from warmer climates (Lancaster et al., 2015), rather than the more locally adapted populations of native species. As such, we hypothesise that increased temperatures will reduce the beneficial effect of complexity for the native species, while increasing the fitness of the range shifter. Finally, we predicted that temperature would have a more significant role in competition at juvenile life stages, given the dependency of growth on temperature and the lack of species niche differentiation at that stage (McPeek, 2004), while biotic resistance would prove more beneficial to native species during their adult life stage, as competition shifts towards a more limited mating opportunity and strong potential impacts of interspecific mating harassment and interference on fitness (Drury et al., 2015).

## 2 METHODS

Permission was not required for fieldwork in this study. This study did not require ethical approval.

### 2.1 Larval mesocosm experiment

Larval mesocosm experiments took place during June and July from 2021 to 2023 (6 mesocosms in 2021, 24 in 2022, and 6 in 2023). Larvae were sampled for these experiments from wild populations in NE Scotland (57.2032, −2.7070) using D‐frame dipnets (~30 cm opening, 1 mm × 1 mm mesh). Intermediate‐instar individuals were selected based on approximate size (head width approx. 2.5 mm‐4 mm); early and late instars were avoided to prevent cohort splitting within the experiment (Nilsson‐Örtman et al., 2014).

**FIGURE 1:**
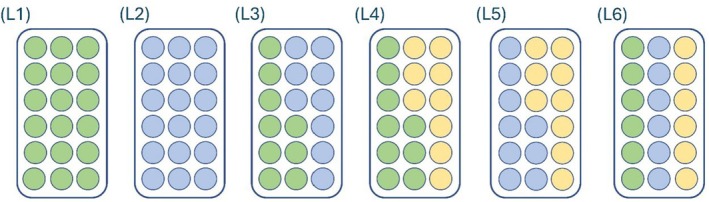
Experimental design for the larval competition experiment. Each treatment began with 18 individuals and was replicated three times per temperature. The numbers beside each treatment (L1–L6) refer to the species composition of each as described in text, but treatments are also colour‐coded here by species for ease of visualisation. Green = *Lestes sponsa* (the focal native species), blue = *Ischnura elegans* (the focal range shifter), and yellow = *Enallagma cyathigerum*, a non‐focal native species.

Six competitive scenarios were devised to test the impacts of increasing community diversity on the demographic outcomes of competition between range shifting (*Ischnura elegans*) and native (*Lestes sponsa*) damselfly larvae. Within each treatment, the total density of larvae was kept constant at 18 individuals, with the species present split evenly across this number (see Figure 1). This approximated *Lestes* larval field densities (approx. 1 larva per 100 mL; Sniegula et al., 2019) and allowed a separation between the predictors of interest and density effects, which have already been extensively studied in damselfly systems (e.g. McPeek & Crowley, 1987). These six competitive larval treatments consisted of the:

L1. *L. sponsa* only (focal native species, intraspecific competition).
L2. *I. elegans* only (range shifter, intraspecific competition).
L3. *L. sponsa* and *I*. *elegans* (focal native interspecific competition with a range shifter).
L4. *L. sponsa* and *E*. *cyathigerum* (focal native interspecific competition with a non‐focal native species).
L5. *L elegans* and *E*. *cyathigerum* (range shifter interspecific competition with non‐focal native).
L6. *L. sponsa*, *I. elegans*, and *E*. *cyathigerum* (multispecies interspecific competition).

(see Figure 1 for a graphical breakdown).

These scenarios allowed investigation of the effects of increasing community complexity on the focal native species, going from intraspecific competition (L1) to interspecific competition (L3–L4) to multispecies competition (L6). It also allowed for comparisons of the competitive effects of range shifters versus non‐range shifters (scenarios L3 and L4) on the focal *L*. *sponsa*. For the range shifter, these treatments similarly allow investigation of the impacts of increasing community diversity on competitive outcomes, from intraspecific competition (L2) to interspecific competition (L3 and L5) to multispecies competition (L6).

Additionally, the above competitive treatments were carried out at both 15 and 20°C, representing current and predicted average summer water temperatures for Scottish freshwater pond habitats (Lancaster et al., 2017), allowing investigation into the potential interactive effects future warming may have on competitive dynamics. Each treatment was replicated three times at each temperature. Competition experiments took place in mesocosms consisting of clear plastic tubs (17 cm × 11 cm × 11.5 cm; Figure 3a), filled with 1.8 L of dechlorinated water. A piece of fake pond weed was also placed in each tub as shelter for the larvae. Each replicate experiment was run in a temperature‐controlled incubator (LMS model 280NP) for 7 days on a 12:12 light: dark cycle; this length of time was sufficient for larvae to moult and grow while experiencing competitive stress, but short enough to prevent individuals from emerging as adults during the experiment. Temperature treatments were randomised temporally, with the incubator being left for 3 days between treatments to fully stabilise at the new temperature. Eighteen *Artemia* brine shrimp were introduced to each experimental unit as live prey food once every 2 days, starting on the first day. This low feeding rate limited food availability, driving competition between larvae. Mesocosms were reorganised randomly within the incubator each day to prevent shelf effects.

Individual larvae were tracked during the experiment via a combination of multiple measures of descriptive morphology (body colour, lamellae shape/number, absence of limbs, etc). This allowed the demographic parameters of larval survival and growth to be measured for individual *L*. *sponsa* and *I*. *elegans*, to determine the response of each species to competition. Larval survival after the 7‐day experimental period was recorded as a binary response variable, with larvae coded 1 for survived and 0 for died (this included individuals that died from starvation/stress, as well as those which had been cannibalised by other larvae). Larval growth over this period was measured by photographing individual larvae at the start and end of each experimental treatment using a Yencam ISH500 camera attached to a Yenway dissecting microscope. The images were then measured using the imaging program Yencam to provide pre‐ and post‐competition head width data (in mm, to 2d.p.), a proxy for overall body size. Larval growth in this experiment was split into two response variables. The first was a binary response of growth (1) versus no growth (0) seen across the experiment's duration, with growth being determined as an increase in head width of at least 0.05 mm (as the degree of measurement error was 0.04 mm). For those that did grow, a second parameter of larval growth rate was calculated as $\frac{\text{Final Head width}\;\left(\mathrm{mm}\right)-\text{Initial Head width}\;\left(\mathrm{mm}\right)}{7}$, the denominator representing the experimental duration in days.

### 2.2 Larval data analysis

Data were analysed on R version 4.2.1 (R Core Team, 2023). For all three demographic parameters of survival, probability of growth and growth rate, models were fit for the larvae of *L*. *sponsa* (focal native species) and *I*. *elegans* (range shifter) independently to test the impacts of novel competition from both the native species and range shifter perspective.

The parameters of survival and probability of growth were modelled using generalised linear mixed models with a binomial error distribution in the glmer() function from the {lme4} package (ver. 1.1–36) (Bates et al., 2015). The main predictor variables for these models were competitive treatment, set as a factor (treatments L1, L3, L4, and L6 for models of *L*. *sponsa*, and treatments L2, L3, L5, and L6 for *I*. *elegans*; see Figure 1), to test the impact of community biodiversity on larval competitive success, and temperature (set as factor, either 15°C or 20°C), to test for impacts of warming on competitive dynamics. Models were fit with these variables both in isolation and together (additive), along with an additional model including an interaction term between them. Initial head width (continuous), year (factor) and day of year (continuous) were all included as additional predictors in all models, along with experimental replicate (factor), which was included as a random variable. Akaike Information Criterion (AIC) (Burnham & Anderson, 2004) values were used to compare goodness of fit between all models. The tbl_regression() function from the {gtsummary} package (ver. 2.1.0) (Sjoberg et al., 2021) was used to obtain global p‐values for the multi‐levelled competitive Treatment and Treatment × Temperature interaction variables.

For individuals that both survived and grew, growth rates were analysed using linear mixed models with the lmer() function of {lme4} (Bates et al., 2015), with growth rates log transformed to better match the assumptions of normality. We again varied the inclusion of and interaction between the main predictor variables of competitive treatment and temperature, and initial head width (continuous), year (factor) and day of year (continuous) were likewise again included as additional predictors in all models, with experimental replicate (factor) again as a random variable. AIC values were used to compare goodness of fit. Post hoc tests for models containing the multileveled Treatment response were carried out using the glht() function from the {multcomp} package (ver. 1.4–26) (Hothorn et al., 2008), which utilises the Tukey HSD method to perform multiple comparisons of means between levels of competitive treatment.

For each demographic parameter, the ggpredict() function from the {ggeffects} package (ver. 2.2.1) (Lüdecke, 2018) was used with the best fit model to predict probability values for the response variable across the range of the other included predictors. These predicted values and their 95% confidence intervals were plotted for each demographic parameter using the {ggplot2} (ver. 3.5.1) (Wickham, 2016) and {ggpubr} (ver. 0.6.0) (Kassambara, 2023) packages.

### 2.3 Adult mesocosm experiment

Adult damselflies were collected via sweep netting at two field sites in the same region of NE Scotland, within the same genetic population as our larval samples (Fitt et al., 2019) (Raemoir Trout fishery 57.069, −2.504; and Loch Cormech 57.0002, −2.5766) in July 2022.

**FIGURE 2:**
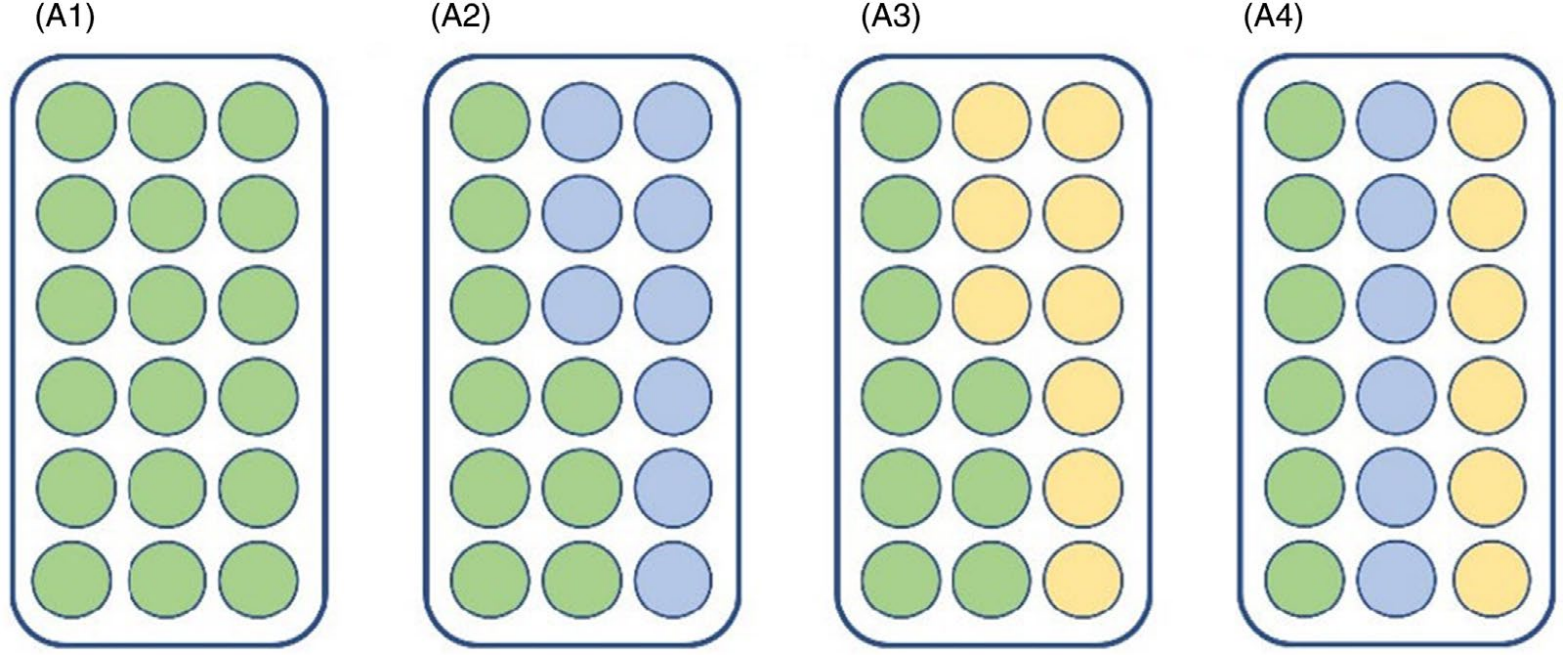
Experimental design for the adult competition experiment. Each treatment again began with 18 individuals and was replicated five times. The numbers beside each treatment (A1–A4) refer to the species composition of each as described in text, but treatments are also colour‐coded here by species. Green = *Lestes sponsa* (the focal native species), blue = *Ischnura elegans* (range shifter), and yellow = *Enallagma cyathigerum*.

Due to the primary goal of this study being the quantification of the impacts of novel range shifter competition on native species, and due to space limitations in outdoor mesocosms, the adult damselfly competition experiment focused primarily on the demographic outcomes of *Lestes sponsa*. To achieve this, we used the same competitive treatments as for the larval *L*. *sponsa* experiments, which consisted of the following:

A1. *L. sponsa* only (focal native species intraspecific competition)
A2. *L. sponsa* and *I. elegans* (focal native and range shifter interspecific competition)
A3. *L. sponsa* and *E. cyathigerum* (focal native and non‐focal native interspecific competition)
A4. *L. sponsa, I. elegans* and *E. cyathigerum* (multispecies interspecific competition)

(see Figure 2 for graphical layout).

As with the larvae, this allowed investigation into the effects of increasing community complexity on focal native life histories (treatments A1, A2/A3 and A4), as well as comparison of the impacts on natives of range shifters versus other native species (treatments A2 and A3). For range shifters, this allowed insights into the effects of increasing resident species diversity on range shifter success, from a single competitor species (treatment A2) to multiple (treatment A4).

Due to the more challenging nature of keeping adult damselflies in the laboratory compared to larvae, this experiment took place in semi‐natural conditions in the Cruickshank Botanic Gardens, Scotland (57.168, −2.104). The damselfly species used in this experiment are found naturally in these gardens (personal observations), confirming the habitat as suitable. While this removed our ability to control for temperature, air temperature was still measured daily during the damselflies' most active period (approx. 2 pm) using a digital thermometer (to 1 d.p.) to be included as a predictor of competitive outcomes.

**FIGURE 3:**
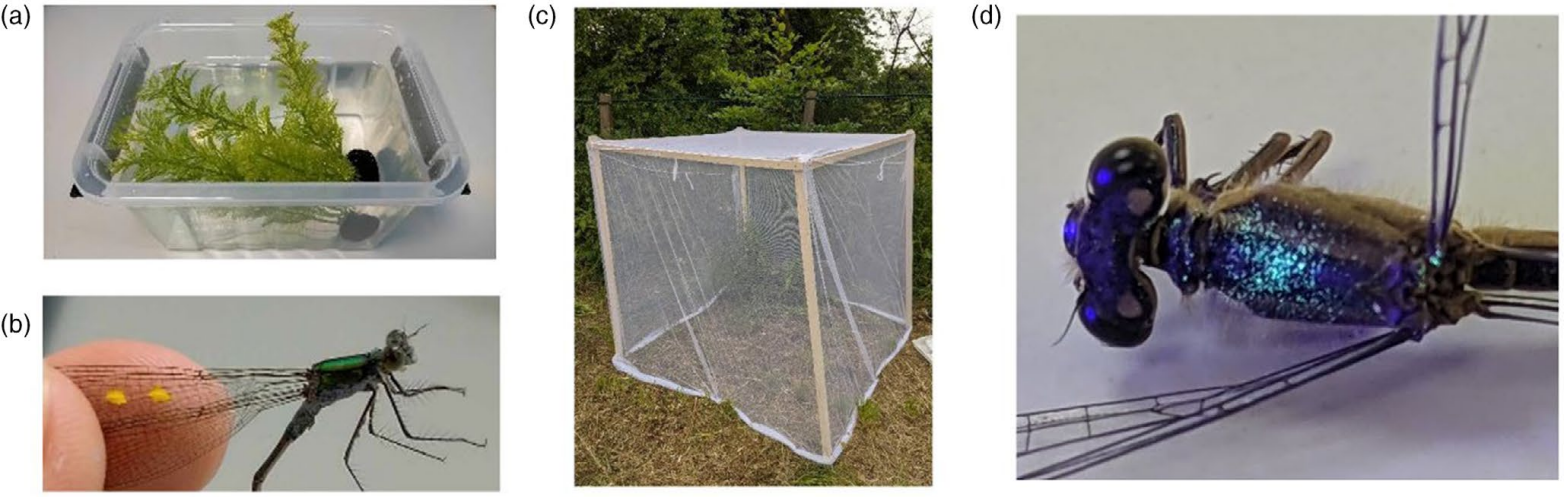
(a) example of a mesocosm used for the larval competition experiment, complete with plastic pond weed. (b) paint dots on the wing of a male *Lestes sponsa*, used for individual identification. (c) One of the mesh cages used for the adult competition experiments. (d) A post‐experiment female *L*. *sponsa* under a UV light showing powder on her thorax, transferred from an interaction with a male *L*. *sponsa*.

Competitive treatments were each replicated five times over the span of 2 weeks. Experiments took place in 1.5m^3^ cages (Figure 3c), consisting of a wooden frame wrapped in mosquito net meshing (mesh size approx. 1.2 mm). This meshing prevented adult damselflies from escaping during the experiment and was left open prior to and in between experimentation to allow prey insects to enter the area. Damselfly densities were kept the same as in the larval experiment (18 individuals per cage). However, adult sex ratios were controlled; set to 5:1 male:female in treatments with either one or three species, and 7:2 in treatments with two species, averaging at a 5:2 male:female ratio across all treatments, matching naturally male‐biased field populations (Dudaniec et al., 2022; Stoks, 2001). Experimental duration was limited to only 24 h to allow multiple mating attempts to occur before the energetic stress of mating harassment impacted individual survival. Before each experiment, damselflies were marked on their wings with nontoxic paint to allow for individual identification (Figure 3b) and were photographed using an Epsom Perfection V37 flatbed scanner at a resolution 600DPI. These scans were used along with the imaging software ImageJ (Schneider et al., 2012) to gain measures of total body size (TL) and hind wing (HW) length (mm, to 2d.p.) for each individual.

Nontoxic fluorescent paint powder was applied to the claspers (caudal abdomen) of male damselflies, after Gosden and Svensson (2009). This allowed transference of paint powder from males to females during attempted copulation and provided a measure of the probability of mating harassment experienced by female damselflies. Powder was applied to males immediately before they entered the mesocosm, and females were examined under UV light for powder transference at the end of the trial 24 h later (see Figure 3d). The presence of powder on females' thorax after the experiment reflected harassment from a male (coded as 1), while absence of powder reflected no harassment (coded 0). Males were colour‐coded by species; however, the results of this experiment are presented below in aggregate due to a limited sample size (but see Supporting Information for discussion of the raw data). This parameter of sexual harassment, along with adult damselfly survival (again coded 1 for survived and 0 for died), formed the response variables for this part of the experiment.

### 2.4 Adult data analysis

As with the larval data, survival of adult damselflies (both *Lestes* and *Ischnura*) was fit to a range of GLMMs with interaction terms included between the main predictor variables of competitive treatment (factor, scenarios A1–A4, as relevant for each species, Figure 2) and temperature (continuous). Additional predictors of hind wing: body size ratio (HW:TL (continuous), which may mediate adult damselfly competition) and sex (factor) were also included, with experimental replicate (factor) again as a random variable. Unlike the larval data, the adult experiment took place entirely within a 2‐week period, so accounting for time lag was unnecessary.

Probability of mating harassment of female *L*. *sponsa* was also modelled as a binomial GLMM, with the same predictor variables as survival (minus the term for sex). AIC values were used to determine model fit. Post hoc tests were carried out for the multileveled Treatment predictor using the glht() function. For interactive models between the categorical treatment and continuous temperature predictors, the emtrends() function of the {emmeans} package (ver. 1.11.0) (Lenth, 2024) was used to estimate and compare the slopes of the temperature effect across levels of competitive treatment, using a 0.95 confidence level. ggpredict and ggplot were again used to estimate and plot survival probabilities for the best fit model.

## 3 RESULTS

We present our results here by species, first outlining the experimental results of the range‐shifting *Ischnura elegans*, followed by our native resident species *Lestes sponsa*. In both cases, we begin by presenting the results of the larval life‐history parameters, followed by adult survival and (in the case of *L*. *sponsa*) mating harassment.

### 3.1 *Ischnura elegans* larvae (range shifter)

#### 3.1.1 Survival

**FIGURE 4:**
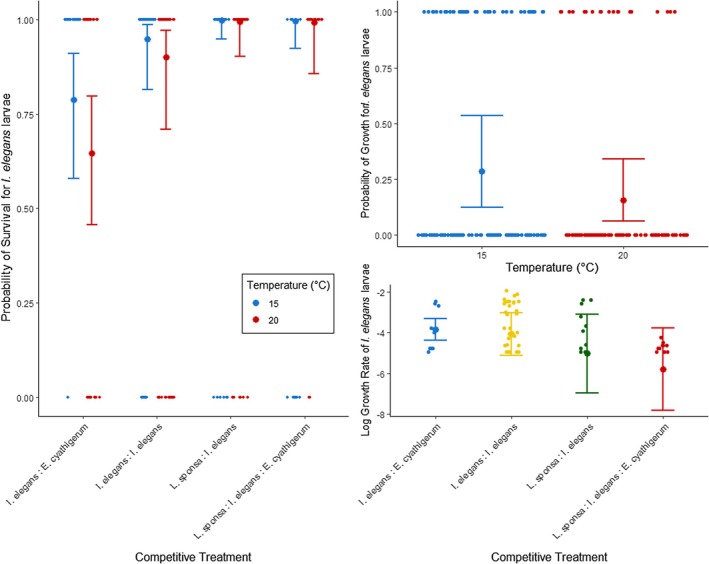
Plotted best fit models for the demographic parameters of survival (left), probability of growth (top right), and growth rate (bottom right) for larval *Ischnura elegans*. Raw data are plotted as smaller points in each graph, jittered to improve visibility, with the predicted probability value for the response variable (along with its corresponding 95% confidence intervals) plotted as the larger dot and its associated error bars.

The probability of *I*. *elegans* larval survival was fit best by an additive model of treatment and temperature (Table S1), with survival improving in colder conditions (Table 1), contrary to our hypothesis. *Ischnura* survival was greatest in treatments that include our focal native species *Lestes sponsa*, both interspecifically and in the multispecies treatment (Figure 4), with post hoc tests revealing no significant difference in survival between these treatments (Table 1). *Ischnura* survival was lowest when in competition with *E*. *cyathigerum*, although post hoc tests revealed this to be statistically comparable to intraspecific survival rates.

**TABLE 1:** The best fit model for *I*. *elegans* larval survival, showing the significant effects of both competitive treatment and temperature, as well as the additional predictors included in the model.

Fixed effect	Estimate	Standard error	*z*‐value	*p*‐value
Intercept	9.9743	3.1859	3.131	**0.002**
Competition treatment				**0.023**
*I. elegans*: *I. elegans*—*I. elegans*: *E. cyathigerum*	1.6024	0.8235	1.946	0.103
*I. elegans*: *L. sponsa*—*I. elegans*: *E. cyathigerum*	4.5156	1.5893	2.841	**0.023**
*I. elegans*: *L. sponsa*: *E. cyathigerum*—*I. elegans*: *E. cyathigerum*	4.3667	1.7315	2.522	**0.047**
*I. elegans*: *L. sponsa*—*I. elegans*: *I. elegans*	2.9132	0.9522	3.06	**0.013**
*I. elegans*: *L. sponsa*: *E. cyathigerum*—*I. elegans*: *I. elegans*	2.7643	1.1048	2.502	**0.047**
*I. elegans L. sponsa*: *E. cyathigerum*—*L. sponsa*: *I. elegans*	−0.1489	0.5287	−0.282	0.778
Temperature	−0.7119	0.3543	−2.009	**0.045**
Initial head width	0.8207	0.4876	1.683	0.092
Year	−3.2318	1.1267	−2.863	**0.004**
Day	−0.0733	0.0211	−3.479	**<0.001**

*Note*: Individual levels of the categorical variable Treatment are shown in italics. The global *p*‐value for the variable is shown in the row above. Significant values are shown in bold.

#### 3.1.2 Probability of growth

The probability of *Ischnura* larvae growing over the course of the experiment was fit best by a model with only temperature as the main predictor (Table S2), with growth overall more likely in colder compared to warmer conditions (Figure 4, Table 2).

**TABLE 2:** Results of the best fit model for the probability of growth in *I*. *elegans* larvae, showing the significant effects of the main predictor of temperature.

Fixed effect	Estimate	Standard error	*z*‐value	*p*‐value
Intercept	0.9385	1.5758	0.596	0.552
Temperature	−0.7827	0.3331	−2.35	**0.019**
Initial head width	−0.6979	0.4532	−1.54	0.124
Year	0.2152	0.5571	0.386	0.839
Day	0.0025	0.008	0.314	0.754

*Note*: Significant values are shown in bold.

#### 3.1.3 Growth rate

For individuals that grew, log transformed growth rate for *Ischnura* larvae was fit best by a model incorporating competition treatment effects, but not temperature (Table S3). The model revealed a significant effect of treatment (Table 3), and post hoc tests revealed a significant decrease in growth between the intraspecific and multispecies treatments (Figure 4).

**TABLE 3:** Results of the best fit model for *I*. *elegans* larvae growth rate.

Fixed effect	Estimate	Standard error	*z*‐value	*p*‐value
Intercept	−2.140	1.712	−1.250	0.220
Treatment				**0.036**
*I. elegans*: *I. elegans*—*I. elegans*: *E. cyathigerum*	−0.2239	0.5342	−0.419	0.675
*L. sponsa*: *I. elegans*—*I. elegans*: *E. cyathigerum*	−1.1781	0.9253	−1.273	0.406
*L. sponsa*: *I. elegans*: *E. cyathigerum*—*I. elegans*: *E. cyathigerum*	−1.944	0.9679	−2.009	0.178
*L. sponsa*: *I. elegans*—*I. elegans*: *I. elegans*	−0.9542	0.5475	−1.743	0.244
*L. sponsa*: *I. elegans*: *E. cyathigerum*—*I. elegans*: *I. elegans*	−1.7201	0.5964	−2.884	**0.024**
*L. sponsa*: *I. elegans*: *E. cyathigerum*—*L. sponsa*: *I. elegans*	−0.7659	0.3494	−2.192	0.142
Initial head width	−1.396	0.264	−5.294	**<0.001**
Year	0.425	0.559	0.759	0.452
Day	0.021	0.011	1.882	0.071

*Note*: Individual levels of the categorical variable Treatment are shown in italics. The global *p*‐value for the variable is shown in the row above. Significant values are shown in bold.

### 3.2 *Ischnura elegans* adult (range shifter)

#### 3.2.1 Treatment survival

**FIGURE 5:**
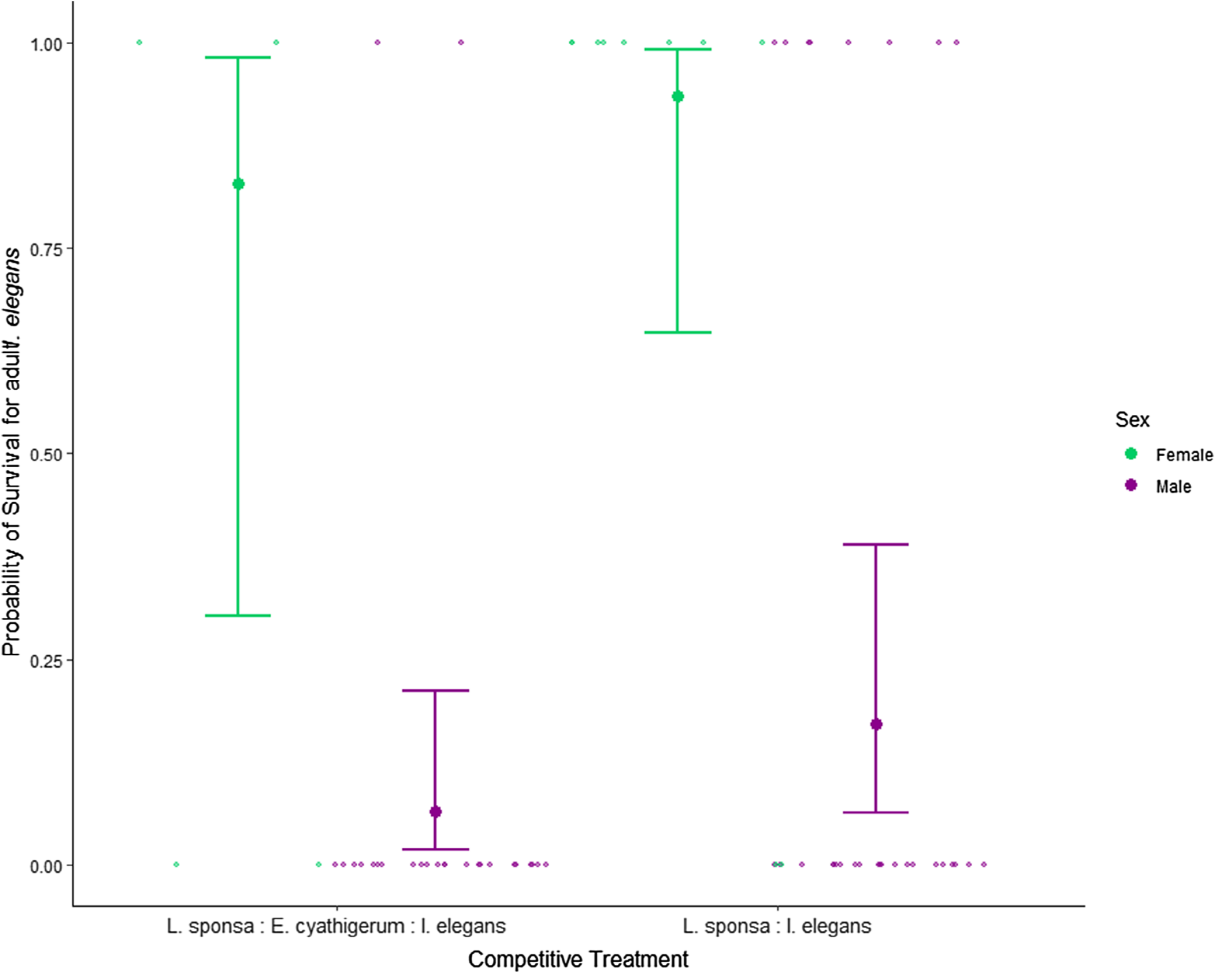
Survival probability of *Ischnura elegans* adults, demonstrating differences in survival between males and females across treatments. Raw data are plotted as the smaller points in each graph, jittered for visibility, with the predicted probability values for adult survival and their corresponding 95% confidence intervals plotted as the larger dots and their associated error bars.

Adult *Ischnura* survival was fit best by a model that included competitive treatment as a primary predictor, as well as terms for both sex and the hindwing: body length ratio (Table S4). Of the three only sex was significant (Table 4), with female survival being much greater than that of males across treatments (Figure 5). The temperature in the adult enclosures ranged from 14.5 to 19°C; this variation did not significantly impact survival and was dropped from the final model.

**TABLE 4:** Results of the best fit model for *I*. *elegans* adult survival, showing the significant effect of sex.

Coefficients	Estimate	Std. error	*z*‐value	*p*‐value
Intercept	19.172	11.438	1.676	0.093
Treatment (*L*. *sponsa*: *I. elegans*)	1.089	0.735	1.482	0.138
HW:TL	032.489	19.326	−1.681	0.093
Sex (Male)	−4.241	1.397	−3.037	**0.002**

*Note*: Significant values are shown in bold.

### 3.3 *Lestes sponsa* larvae (native species)

#### 3.3.1 Survival

**FIGURE 6:**
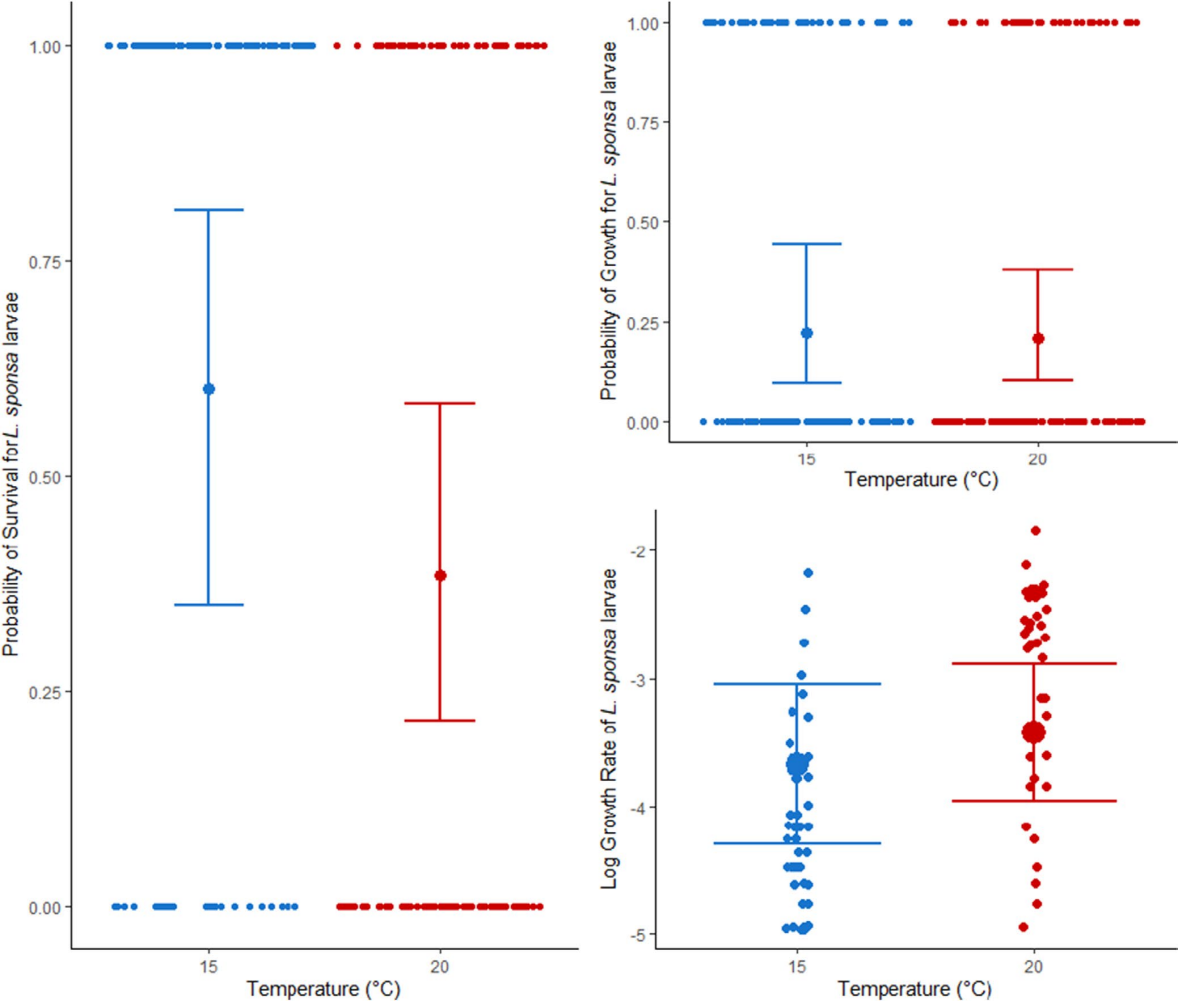
Plotted best fit models for the demographic parameters of survival (left), probability of growth (top right), and growth rate (bottom right) for larval *Lestes sponsa*. As above, raw data are plotted as smaller points in each graph, with jitter added among the points for clarity. The predicted probability value for the response variable, along with its corresponding 95% confidence intervals, is plotted as the larger dots and their associated error bars.

The survival probability of *L*. *sponsa* larvae was explained best by a model with a weakly significant temperature effect as the primary fixed effect (Table S5), with survival being generally more likely in colder conditions (Figure 6, Table 5). Unlike *I*. *elegans*, the survival of *Lestes* did not differ between competitive treatments.

**TABLE 5:** Results of the best fit model for *L*. *sponsa* larval survival, showing the significant effect of initial head width as well as the weakly significant effect of the main predictor temperature.

Fixed effect	Estimate	Standard error	*z*‐value	*p*‐value
Intercept	−1.56699	3.96467	−0.395	0.693
Temperature	−0.88094	0.50411	−1.748	0.081
Initial head width	1.82596	0.48389	3.774	**<0.001**
Year	0.37776	0.59682	0.633	0.527
Day	−0.02292	0.02515	−0.911	0.362

*Note*: Significant values are shown in bold.

#### 3.3.2 Probability of growth

The probability of growth occurring in *Lestes* larvae was fit best by a model with just temperature as the primary predictor (Table S6). Despite this, neither temperature nor any of the other predictor variables showed any significant effect on the probability of growth in *Lestes* larvae (Table 6). Competitive treatment again had no effect on the outcome of this parameter (Figure 6).

**TABLE 6:** Results of the best fit model for the probability of growth in *L*. *sponsa* larvae, showing lack of significance across predictors.

Fixed effect	Estimate	Standard error	*z*‐value	*p*‐value
Intercept	−3.59141	3.79549	−0.946	0.344
Temperature	−0.08401	0.47111	−0.178	0.858
Initial head width	−0.02619	0.44655	−0.059	0.953
Year	0.73386	0.59304	1.237	0.216
Day	0.01358	0.02389	0.568	0.57

#### 3.3.3 Growth rate

The log transformed growth rate of *Lestes* larvae was explained best by a model with just temperature as the main predictor (Table S7). Despite this, only initial larval head width returned as a significant determinant of *Lestes* larval growth rate (Table 7, Figure 6).

**TABLE 7:** Results of the best fit model for *L*. *sponsa* larval growth rate.

Fixed effect	Estimate	Standard error	*z*‐value	*p*‐value
Intercept	0.533	2.373	0.225	0.823
Temperature	0.249	0.252	0.992	0.325
Initial head width	−0.833	0.267	−3.114	**0.002**
Year	−0.123	0.369	−0.334	0.739
Day	−0.008	0.015	−0.515	0.608

*Note*: Significant values are shown in bold.

### 3.4 *Lestes sponsa* adults

#### 3.4.1 Survival

**FIGURE 7:**
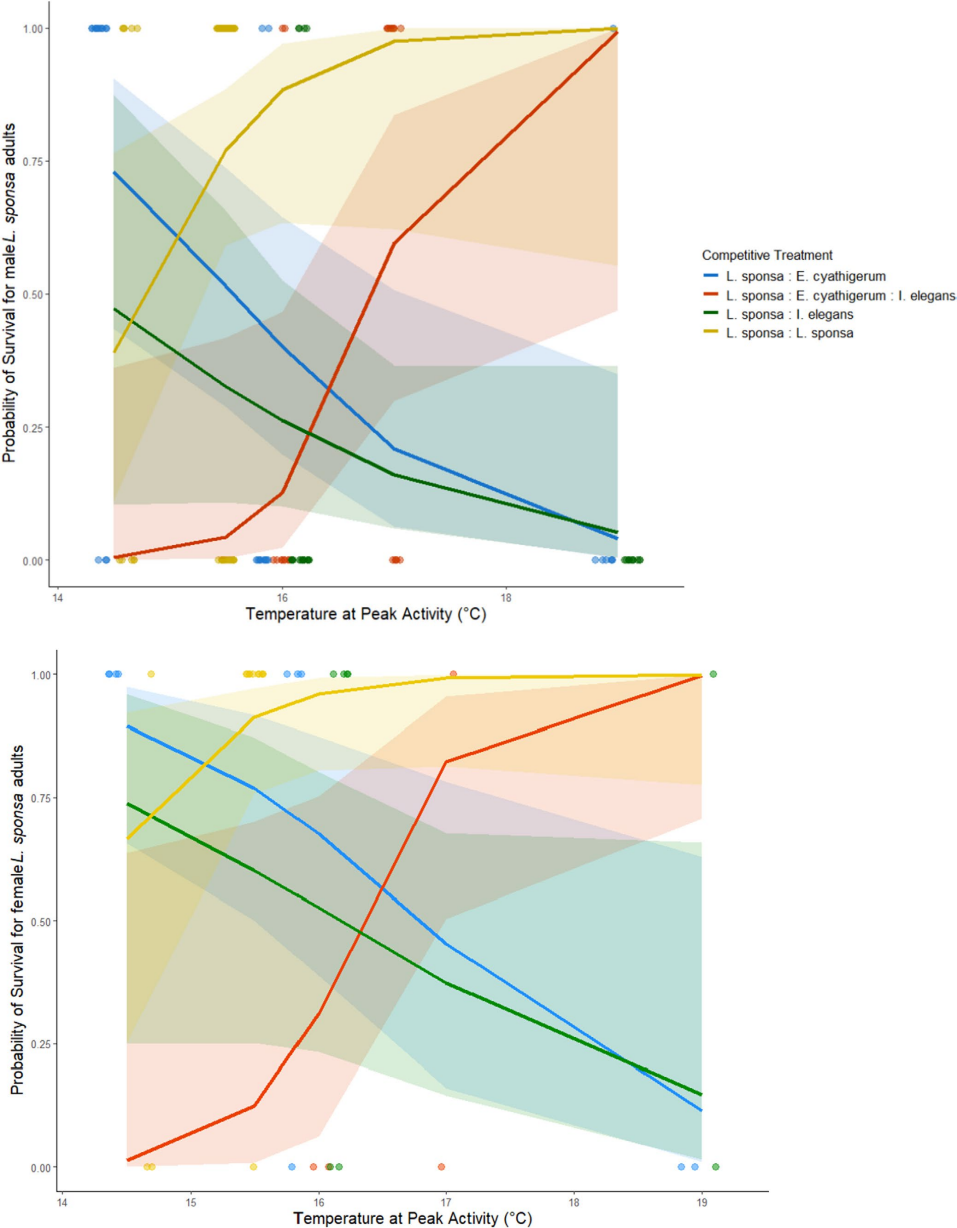
Survival probability of *Lestes sponsa* adult males (above) and females (below) showing the interaction of temperature and treatment. Trendlines show the curve of predicted survival for each treatment, with 95% model confidence limits shown in the corresponding colour.

Probability of survival in *Lestes* adults was described best by an interactive model of treatment and temperature (Table S8). When in pairwise interspecific competition, *Lestes* do better at lower temperatures, with survival dropping as temperature increases (Table 9). However, in both the intraspecific and multispecies treatments, *Lestes* survival greatly increases with temperature, to the point of almost complete survival in the intraspecific treatment (Figure 7). The best fit model also included an effect for sex, with survival being lower in males compared to females (Table 8).

**TABLE 8:** Results of the best fit model for *L*. *sponsa* adult survival.

Fixed effect	Estimate	Standard error	*z*‐value	*p*‐value
Intercept	15.608	5.652	2.762	**0.005**
Treatment				**0.006**
*L. sponsa*: *E. cyathigerum—L. sponsa*: *E. cyathigerum*: *I. elegans*	−3.254	1.179	−2.761	**0.029**
*L. sponsa*: *E. cyathigerum—L. sponsa*: *I. elegans*	−0.309	0.557	−0.556	0.945
*L. sponsa*: *E. cyathigerum—L. sponsa*: *L. sponsa*	−2.585	1.014	−2.551	**0.052**
*L. sponsa*: *E. cyathigerum*: *I. elegans—L. sponsa*: *I. elegans*	2.945	1.203	2.448	0.068
*L. sponsa*: *E. cyathigerum*: *I. elegans—L. sponsa*: *L. sponsa*	0.669	1.452	0.461	0.967
*L. sponsa*: *I. elegans*—*L. sponsa*: *L. sponsa*	−2.276	1.042	−2.184	0.127
Temperature	−0.9292	0.3559	−2.611	**0.009**
Sex (Male)	−1.1431	0.4894	−2.336	**0.019**
Treatment × Temperature				**0.011**

*Note*: Individual levels of the categorical Treatment variable are shown in italics. Significant values are shown in bold.

**TABLE 9:** Estimated slopes of the interactive effect of the continuous Temperature variable across levels of the categorical Treatment variable on adult *L*. *sponsa* survival, averaged over levels of Sex.

Treatment	Temperature trend	SE	Lower confidence limits	Upper confidence limits
*L. sponsa*: *E. cyathigerum*	−0.929	0.356	−1.627	−0.232
*L. sponsa*: *E. cyathigerum*: *I. elegans*	2.325	1.12	0.129	4.521
*L. sponsa*: *I. elegans*	−0.62	0.429	−1.461	0.221
*L. sponsa*: *L. sponsa*	1.656	0.945	−0.197	3.509

#### 3.4.2 Mating harassment

**FIGURE 8:**
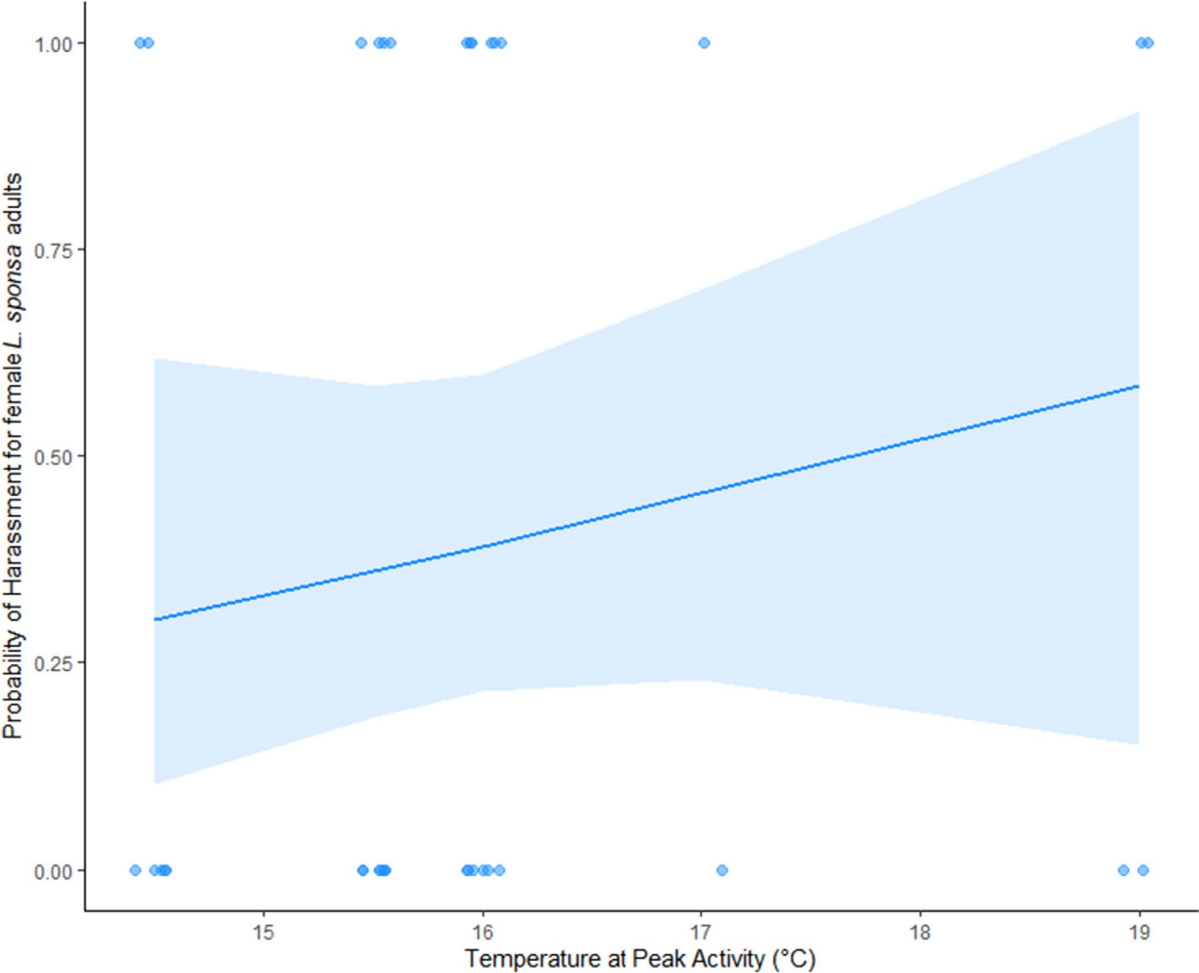
Plotted best fit model for mating harassment experienced by female adult *Lestes sponsa*.

The degree of mating harassment experienced by adult female *Lestes sponsa* was fitted best by a model of exclusively temperature (Table S9). This temperature effect was, however, non‐significant (Table 10, Figure 8).

**TABLE 10:** Results of the best fit model for the probability of mating harassment experienced by adult female *L*. *sponsa*, showing a lack of significance in the main temperature effect.

Fixed effect	Estimate	Standard error	*z*‐value	*p*‐value
Intercept	−4.646	5.809	−0.8	0.424
Temperature	0.263	0.359	0.733	0.464

## 4 DISCUSSION

The propensity for communities with increased biological complexity to inhibit the fitness/success of nonnative species, termed biotic resistance, is a prevalent mechanism in the world of invasion biology (Beaury et al., 2020). Evidence of biotic resistance impacting the success of novel invasive species is widespread; however, its potential role in determining the outcome of novel competition stemming from range shift events is only beginning to garner more attention, with most work in the area so far remaining conceptual (Guo et al., 2012) or literature based (Sorte et al., 2010; Wallingford et al., 2020), and lacking explicit testing. In this study, our results show that increasing community complexity negatively impacts larval growth of the range shifting damselfly *Ischnura elegans* (Figure 4), providing early experimental evidence of competition‐based biotic resistance impeding the success of a range shifting species. While our results reflect only a specific temporal window of response (the response of mid‐instar larvae across a week of experimental competitive interactions), this short experimental exposure is nonetheless sufficient to capture the impacts of biotic resistance on a non‐native range shifting species. However, these impacts are not constant across all life history parameters; while increased complexity does negatively affect growth, it does not directly impact the survival of our range shifting species (Figure 4). This implies that while competitive interactions may not prevent range shifters from establishing within a resident community, they may still act to constrain their abundance and long‐term population success (Levine et al., 2004).

Our results further demonstrate that both the strength and the directionality of this resistance are heavily dependent on organism life stage, with increased community complexity negatively impacting range shifter growth rate in the larval stage, while having no significant impact on adult fitness (Figure 5). Such life stage dependence of biotic resistance is not uncommon in the realm of invasive species (Rius et al., 2014), especially when complex life cycles involve an ontogenetic shift in habitat, which can drastically alter both the environmental and competitive landscape (Nakazawa, 2015). Diverging competitive abilities and strategies across a life cycle can be impacted by carry‐over effects between life stages (Brodin, 2009), altering the overall outcomes of competition between range shifting and resident species. Changes in habitat between larval and adult life stages also bring with it new environmental stressors and tolerances (which may again be impacted by the previous life stage (Harabiš et al., 2012)), potentially mediating the dynamics of coexistence via indirect impacts on competitive outcomes. This is reflected in our results, in which we find thermal effects limiting success in the larval life stage but not in adults (Figures 4 and 5). This life stage dependence of biotic resistance on non‐resident species adds nuance to the outcomes of range shifter competition, as competitive disadvantages in one life stage may be compensated for in another.

Functional similarity between novel and resident species may also play a role in determining the success and establishment of novel species (Conti et al., 2018), although evidence can be mixed (Guo et al., 2024). Functionally similar species are expected to experience stronger competitive interactions for space and resources, impacting alien success (Conti et al., 2018). We find evidence of this here, with the range‐shifting *Ischnura elegans* performing poorly when competing against the functionally similar resident *Enallagma* species, compared to the more dissimilar *Lestes* (Figure 4). Given the links between functional similarity and phylogenetic distance (Cadotte et al., 2013), phylogeny too may prove to be a relevant factor in determining range‐shifter success, although this will require more explicit testing. However, the ability of functional dissimilarity to mediate biotic resistance is itself influenced by environmental stress (Conti et al., 2018), further underlining the importance of investigating these competition dynamics across environmental gradients, especially given anticipated shifts in temperature and weather patterns resulting from contemporary climate change.

In this study we investigated not only how biotic resistance could restrict the success of a range‐shifting species, but also how it could positively impact the fitness of an individual resident species by sheltering it from novel competition, an aspect of biotic resistance that is often overlooked. We find resident *Lestes* to be unaffected by any competitive treatment at the larval stage (Figure 6), but show improved survival with increasing community complexity and temperature at the adult stage (Figure 7), again proving the impacts of biotic resistance to be both environment and life stage dependent. Interestingly, we find a strong contrast in the directionality of effects between range‐shifting and resident species; while range shifters experienced negative effects of increasing complexity as larvae and none as adults, the inverse is true for resident *Lestes*. As with range shifters, this difference in competitive strength between life stages for resident species likely arises due to changes in niche partitioning or interspecific differences in stage‐specific environmental sensitivities (Komoroske et al., 2014), as evidenced by the significant effect of temperature on adult *Lestes* survival (Figure 7), a dependence that is not mirrored in any measured traits of larval fitness (Figure 6). Interestingly, this interaction of temperature with community complexity serves to provide greater survival benefits to adult *Lestes* in warmer ambient conditions, the opposite of our initial hypothesis. This effect is likely explained by the increased ambient temperature driving stronger competitive interactions between the more active *Ischnura* and *Enallagma* competitors (Amarasekare & Coutinho, 2014), allowing the less competitive *Lestes* to escape competitive harassment (but see Supporting Information for a more detailed discussion of the specific ecological and sexual interactions). These results thus highlight the importance of investigating biotic interactions in the context of more biologically complex, multispecies environments, as it may reveal indirect mechanisms of competition avoidance that would go unnoticed in a simpler two‐species system.

An additional finding of our study was the discovery that competition between range shifters and resident species need not always be a zero‐sum game. Despite the fact that our mesocosm experiments were explicitly designed to drive competition between species and individuals by restricting resource/nutritional availability, we found multiple scenarios that resulted in either no net loss of recorded fitness parameters for both range shifters and residents (neutral effects), or which actively provided benefit to one without causing detriment to the other (facilitation). This is especially remarkable in the context of damselflies, which commonly display cannibalistic tendencies under nutritional stress (De Block & Stoks, 2004).

Interestingly, these facilitative effects can go both ways too. For the range‐shifting *Ischnura elegans*, the presence of resident *Lestes* increased larval survival compared to both interspecific competition with *Enallagma cyathigerum* and intraspecific scenarios (Figure 4), likely resulting from greater functional dissimilarity and subsequent niche partitioning between *Ischnura* and *Lestes* larvae. Such life stage‐dependent facilitative interactions have also been found in range‐shifting experiments using montane trees (Ettinger & HilleRisLambers, 2017) and demonstrate that species‐specific interactions with resident species (Bulleri et al., 2016) may facilitate the establishment and expansion of a range shifter (HilleRisLambers et al., 2013). Furthermore, as mentioned above, adult *Lestes sponsa* undergo an environmental (temperature) dependent shift from competition to facilitation in more complex multispecies communities, with the presence of the range‐shifting *Ischnura* serving to alleviate the competitive stress experienced by *Lestes* against a native competitor (Figure 7). This demonstrates that while resident species may suffer under novel competition with range shifters in an isolated, two‐species system (Fitt & Lancaster, 2017), the nature of this interaction may change across more environmentally and biologically complex scenarios, allowing range‐shifting species to provide off‐label benefits to resident species, facilitating coexistence.

Overall, our results show support for the relevance of biotic resistance as a mechanism driving competitive dynamics in the context of range‐shifting species. However, nuances of our results highlight its potential complexities. We demonstrate that the impacts of biotic resistance, both to limit range‐shifter success and improve individual resident species fitness when faced with novel competition, are highly context‐dependent, with environmental conditions, in particular temperature, serving to influence the directionality of interactions between competition and facilitation. However, more work is yet needed on the role of community functional similarity/dissimilarity in these interactions, which may mediate the impacts of biotic resistance if ecological differences between species allow escape from competition via niche partitioning. Finally, we demonstrate the importance of life stage in determining the impact of biotic resistance on the outcome of novel competitive interactions, and how interspecific variation in demographic sensitivities of population growth across life stages can cause asymmetrical responses between species, leading to potential coexistence and even facilitation between resident and range‐shifting species.

## AUTHOR CONTRIBUTIONS

Michael O'Connor and Lesley T. Lancaster conceived the ideas and designed methodology; Michael O'Connor collected and analysed the data; Michael O'Connor and Lesley T. Lancaster wrote the manuscript.

## CONFLICT OF INTEREST STATEMENT

The authors declare no conflicts of interest.

## Supporting information


**Table S1.** Comparison of fit for generalised linear mixed models of the probability of survival of *I. elegans* larvae.
**Table S2.** Comparison of fit for generalised linear mixed models of the probability of growth of *I. elegans* larvae.
**Table S3.** Comparison of fit for linear mixed models of log transformed growth rate of *I. elegans* larvae.
**Table S4.** Comparison of fit for linear mixed models for survival of *I. elegans* adults.
**Table S5.** Comparison of fit for generalised linear mixed models of the probability of survival of *L. sponsa* larvae.
**Table S6.** Comparison of fit for generalised linear mixed models of the probability of growth of *L. sponsa* larvae.
**Table S7.** Comparison of fit for linear mixed models of log transformed growth rate of *L. sponsa* larvae.
**Table S8.** Comparison of fit for generalised linear mixed models of the probability of survival of *L. sponsa* adults.
**Table S9.** Comparison of fit for generalised linear mixed models of the probability of mating harassment experienced by adult female *L. sponsa*.
**Figure S1.** Raw data for the attempted copulations of male damselflies per female *Lestes sponsa*.

## Data Availability

Data available from the Zenodo data repository: https://doi.org/10.5281/zenodo.15065581 (O'Connor, 2025).
