# Supplementary material for: Guild diversity impacts demographic outcomes of novel species interactions following range shifts
Source: J Anim Ecol. 2025 Jul 25;94(10):2062–78. doi: 10.1111/1365-2656.70108 (PMC12484426; doi:10.1111/1365-2656.70108)
Supplement: Supplementary file 1 — Table S1. Comparison of fit for generalised linear mixed models of the probability of survival of I. elegans larvae. Table S2. Comparison of fit for generalised linear mixed models of the probability of growth of I. elegans larvae. Table S3. Comparison of fit for linear mixed models of log transformed growth rate of I. elegans larvae. Table S4. Comparison of fit for linear mixed models for survival of I. elegans adults. Table S5. Comparison of fit for generalised linear mixed models of the probability of survival of L. sponsa larvae. Table S6. Comparison of fit for generalised linear mixed models of the probability of growth of L. sponsa larvae. Table S7. Comparison of fit for linear mixed models of log transformed growth rate of L. sponsa larvae. Table S8. Comparison of fit for generalised linear mixed models of the probability of survival of L. sponsa adults. Table S9. Comparison of fit for generalised linear mixed models of the probability of mating harassment experienced by adult female L. sponsa. Figure S1. Raw data for the attempted copulations of male damselflies per female Lestes sponsa. [file JANE-94-2062-s001.docx]

Supplementary Material

Table S1. Comparison of fit for generalised linear mixed models of the probability of survival of *I. elegans* larvae. Best fit model highlighted in bold.

| **Model** | **AIC** |
| --- | --- |
| Probability of Survival ~ Treatment*Temperature + Initial head width + Year + Day + (1\|Replicate) | 275.142 |
| Probability of Survival ~ Treatment + Temperature + Initial head width + Year + Day + (1\|Replicate) | **273.209** |
| Probability of Survival ~ Treatment + Initial head width + Year + Day + (1\|Replicate) | 275.319 |
| Probability of Survival ~ Temperature + Initial head width + Year + Day + (1\|Replicate) | 281.553 |

Table S2. Comparison of fit for generalised linear mixed models of the probability of growth of *I. elegans* larvae. The best fit model is highlighted in bold.

| **Model** | **AIC** |
| --- | --- |
| Probability of Survival ~ Treatment*Temperature + Initial head width + Year + Day + (1\|Replicate) | 286.032 |
| Probability of Survival ~ Treatment + Temperature + Initial head width + Year + Day + (1\|Replicate) | 282.344 |
| Probability of Survival ~ Treatment + Initial head width + Year + Day + (1\|Replicate) | 286.221 |
| Probability of Survival ~ Temperature + Initial head width + Year + Day + (1\|Replicate) | **279.330** |

Table S3. Comparison of fit for linear mixed models of log transformed growth rate of *I. elegans* larvae. Best fit model highlighted in bold.

| **Model** | **AIC** |
| --- | --- |
| Growth Rate ~ Treatment*Temperature + Initial head width + Year + Day + (1\|Replicate) | 168.512 |
| Growth Rate ~ Treatment + Temperature + Initial head width + Year + Day + (1\|Replicate) | 170.412 |
| Growth Rate ~ Treatment + Initial head width + Year + Day + (1\|Replicate) | **168.137** |
| Growth Rate ~ Temperature + Initial head width + Year + Day + (1\|Replicate) | 174.457 |

Table S4. Comparison of fit for linear mixed models for survival of *I. elegans* adults. Best fit model highlighted in bold.

| **Model** | **AIC** |
| --- | --- |
| Probability of Survival ~ Treatment*Temperature + HW:TL + Sex + (1\|Replicate) | 72.743 |
| Probability of Survival ~ Treatment*Temperature + HW:TL + (1\|Replicate) | 81.880 |
| Probability of Survival ~ Treatment*Temperature + Sex + (1\|Replicate) | 72.933 |
| Probability of Survival ~ Treatment*Temperature + (1\|Replicate) | 83.181 |
| Probability of Survival ~ Treatment + Temperature + HW:TL + Sex + (1\|Replicate) | 72.181 |
| Probability of Survival ~ Treatment + Temperature + HW:TL+ (1\|Replicate) | 82.737 |
| Probability of Survival ~ Treatment + Temperature + Sex + (1\|Replicate) | 73.428 |
| Probability of Survival ~ Treatment + Temperature + (1\|Replicate) | 83.784 |
| Probability of Survival ~ Treatment + HW:TL + Sex + (1\|Replicate) | **70.545** |
| Probability of Survival ~ Treatment + HW:TL + (1\|Replicate) | 80.923 |
| Probability of Survival ~ Treatment + Sex + (1\|Replicate) | 71.657 |
| Probability of Survival ~ Treatment + (1\|Replicate) | 82.071 |
| Probability of Survival ~ Temperature + HW:TL + Sex + (1\|Replicate) | 71.820 |
| Probability of Survival ~ Temperature + HW:TL + (1\|Replicate) | 87.438 |
| Probability of Survival ~ Temperature + Sex + (1\|Replicate) | 75.632 |
| Probability of Survival ~ Temperature + (1\|Replicate) | 87.290 |

Table S5. Comparison of fit for generalised linear mixed models of the probability of survival of *L. sponsa* larvae. Best fit model highlighted in bold.

| **Model** | **AIC** |
| --- | --- |
| Probability of Survival ~ Treatment*Temperature + Initial head width + Year + Day + (1\|Replicate) | 292.061 |
| Probability of Survival ~ Treatment + Temperature + Initial head width + Year + Day + (1\|Replicate) | 287.923 |
| Probability of Survival ~ Treatment + Initial head width + Year + Day + (1\|Replicate) | 291.651 |
| Probability of Survival ~ Temperature + Initial head width + Year + Day + (1\|Replicate) | **286.401** |

Table S6. Comparison of fit for generalised linear mixed models of the probability of growth of *L. sponsa* larvae. Best fit model highlighted in bold.

| **Model** | **AIC** |
| --- | --- |
| Probability of Growth ~ Treatment*Temperature + Initial head width + Year + Day + (1\|Replicate) | 329.888 |
| Probability of Growth ~ Treatment + Temperature + Initial head width + Year + Day + (1\|Replicate) | 327.141 |
| Probability of Growth ~ Treatment + Initial head width + Year + Day + (1\|Replicate) | 325.815 |
| Probability of Growth ~ Temperature + Initial head width + Year + Day + (1\|Replicate) | **323.433** |

Table S7. Comparison of fit for linear mixed models of log transformed growth rate of *L. sponsa* larvae. Best fit model highlighted in bold.

| **Model** | **AIC** |
| --- | --- |
| Probability of Growth ~ Treatment*Temperature + Initial head width + Year + Day + (1\|Replicate) | 187.427 |
| Probability of Growth ~ Treatment + Temperature + Initial head width + Year + Day + (1\|Replicate) | 190.471 |
| Probability of Growth ~ Treatment + Initial head width + Year + Day + (1\|Replicate) | 187.783 |
| Probability of Growth ~ Temperature + Initial head width + Year + Day + (1\|Replicate) | **186.608** |

Table S8. Comparison of fit for generalised linear mixed models of the probability of survival of *L. sponsa* adults. Best fit model highlighted in bold.

| **Model** | **AIC** |
| --- | --- |
| Probability of Survival ~ Treatment*Temperature + HW:TL + Sex + (1\|Replicate) | 217.556 |
| Probability of Survival ~ Treatment*Temperature + HW:TL + (1\|Replicate) | 220.462 |
| Probability of Survival ~ Treatment*Temperature + Sex + (1\|Replicate) | **216.269** |
| Probability of Survival ~ Treatment*Temperature + (1\|Replicate) | 220.154 |
| Probability of Survival ~ Treatment + Temperature + HW:TL + Sex + (1\|Replicate) | 222.718 |
| Probability of Survival ~ Treatment + Temperature + HW:TL+ (1\|Replicate) | 225.767 |
| Probability of Survival ~ Treatment + Temperature + Sex + (1\|Replicate) | 221.641 |
| Probability of Survival ~ Treatment + Temperature + (1\|Replicate) | 225.132 |
| Probability of Survival ~ Treatment + HW:TL + Sex + (1\|Replicate) | 224.458 |
| Probability of Survival ~ Treatment + HW:TL + (1\|Replicate) | 227.070 |
| Probability of Survival ~ Treatment + Sex + (1\|Replicate) | 223.072 |
| Probability of Survival ~ Treatment + (1\|Replicate) | 226.832 |
| Probability of Survival ~ Temperature + HW:TL + Sex + (1\|Replicate) | 219.923 |
| Probability of Survival ~ Temperature + HW:TL + (1\|Replicate) | 222.793 |
| Probability of Survival ~ Temperature + Sex + (1\|Replicate) | 218.801 |
| Probability of Survival ~ Temperature + (1\|Replicate) | 222.017 |

Table S9. Comparison of fit for generalised linear mixed models of the probability of mating harassment experienced by adult female *L. sponsa*. Best fit model highlighted in bold.

| **Model** | **AIC** |
| --- | --- |
| Probability of Harassment ~ Treatment*Temperature + HW:TL + (1\|Replicate) | 61.825 |
| Probability of Harassment ~ Treatment*Temperature + (1\|Replicate) | 59.563 |
| Probability of Harassment ~ Treatment + Temperature + HW:TL + (1\|Replicate) | 58.927 |
| Probability of Harassment ~ Treatment + Temperature + (1\|Replicate) | 57.459 |
| Probability of Harassment ~ Treatment + HW:TL + (1\|Replicate) | 57.090 |
| Probability of Harassment ~ Treatment + (1\|Replicate) | 55.893 |
| Probability of Harassment ~ Temperature + HW:TL + (1\|Replicate) | 55.094 |
| Probability of Harassment ~ Temperature + (1\|Replicate) | **53.602** |

Discussion of Ecological Relationships

Biotic Resistance on Range Shifter Success

The premise of biotic resistance states that communities with greater biodiversity will be more resilient against an invading species, as a more biologically complex, competitive environment should have a more negative competitive impact on the fitness of the invader (Levine et al., 2004). By this logic, we predicted that the range shifting *Ischnura elegans* would see a reduced demographic success in more complex, multispecies competitive treatments compared to simpler two-species interspecific or even one species intraspecific scenarios. However, experimental testing of this hypothesis returned mixed results. *Ischnura* larval survival was very high in complex multispecies treatments, but also in interspecific treatments with just *L. sponsa*, compared to interspecific competition with the non-focal resident species *Enallagma cyathigerum* as well as intraspecific scenarios (Table 1, Fig. 4). This suggests that *Ischnura* survival is impacted less by competitive complexity and more so benefitted by the presence of *Lestes*, possibly due to differing foraging behaviours between species. Interestingly, *L. sponsa* survival was not reciprocally negatively impacted by *I. elegans*, suggesting that direct competitive asynchrony with *Lestes* is not the primary driver of benefit to *Ischnura.*

Trade-offs between activity levels and predation risk are well documented across many organisms (Duriez et al., 2005; Martin et al., 2000; Wooster and Sih, 1995), and damselflies are no exception (Schaffner and Anholt, 1998), with greater activity levels typically yielding greater foraging returns while incurring larger penalties with regards to predation and survival (Brodin and Johansson, 2004). Both *Ischnura* and *Enallagma* are pond damselflies of the family Coenagrionidae, a group known to display strong ecological conservatism between taxa (Svensson, 2012). Consequently, *Ischnura* and *Enallagma* are known to lack niche differentiation at the larval stage (McPeek, 2004), with both species utilising and competing for pond vegetation as sheltered perches, preferring to minimise activity levels to reduce the risk of predation. This shared evolutionary history in both habitat usage and predator defence leads to an overlapping requirement for a limited perching space, resulting in strong competition between these species, and even between individuals of the same species in intraspecific competitive scenarios. Given the often cannibalistic nature of competition between damselfly larvae (Start et al., 2017), this potentially explains the reduced survival seen for *Ischnura* larvae in these treatments. Comparatively, *Lestes sponsa* is a much more active larval forager, seeking out food while utilising shelter to a much lesser degree, especially in the absence of predators (Stoks, 1999). This difference in behaviours may effectively partition the microhabitat between species, limiting the need for direct competitive interactions between individuals, facilitating coexistence. Further work based on these results, and incorporating different levels of taxonomic relatedness, could highlight how the success of a range shifting species can thus be influenced by shared evolutionary history with resident native species, as phylogenetic distance may impact the strength of competition experienced. This relationship, however, may be further impacted by the presence of predators, as a stronger pressure to selectively and plasticly reduce activity rate (Strobbe et al., 2011) will limit the capacity for niche differentiation between competitive range shifting and native species.

While these differing foraging strategies may facilitate greater *Ischnura* survival, they can have more negative consequences for other important life history parameters. As a more active forager, the presence of *Lestes* serves to reduce the availability of food for other species, lowering *Ischnura* growth rate (Fig. 4). However, if this were the only driver of growth rate, we would expect for *Ischnura* growth to be lowest in all treatments in which *Ischnura* compete with *Lestes*. Instead, we see a significant reduction in growth rate from intraspecific levels only in the multispecies treatment (Table 3). This suggests that while *Lestes* may indeed be strong larval competitors with pronounced impacts on *Ischnura* growth rates, the presence of additional species compounds this effect, supporting the idea of biotic resistance having a more negative impact on range shifter demographics compared to less biologically complex competitive situations.

By contrast, competition between adult damselflies yields no suggestion of biotic resistance impacting range shifter success at this life stage. Adult *Ischnura* are unaffected by competitive treatment, showing very similar survival rates in interspecific vs multispecies scenarios (Fig. 5). This suggests that adult *Ischnura*, unlike their larvae, are more resistant to competitive differences between species, remaining relatively unaffected by increasing levels of biological complexity and overall community competitive strength. Together these results suggest that biotic resistance can indeed impact the likelihood of success for a range shifting species, but that the strength of competition experienced may depend heavily on behavioural differences between species of competitors, as well as the life stage involved. *Ischnura* entering more biologically complex habitats may suffer lower larval growth rates, but this reduction in fitness may be compensated by increases in survival, if mesocosm conditions are representative of competitive conditions in the wild.

Temperature Effects on Range Shifter Success

The range shifting *Ischnura elegans* was predicted to perform more favourably in warmer conditions, with its broad distribution (Willink et al., 2024) and its more recent arrival into higher latitude communities from warmer climates (Lancaster et al., 2015) lending it a broader range of thermal tolerances. This competitive advantage, especially over the locally adapted resident species, was anticipated to result in greater demographic success for the range shifter, to the point of mitigating the expected negative impacts of biotic resistance. However, we found that with regards to both survival and the probability of growth, range shifters did better in colder environments, with no interaction of temperature by competition appearing in the best fit models (Fig. 4). Warmer conditions increases the energy availability for ectothermic larvae, which may increase activity and consequently competition between individuals, leading to lower survival and growth (Nilsson-Örtman et al., 2014). However, a more likely explanation for these results is that *Ischnura* too has undergone local adaptation to the colder conditions in higher latitude habitats along their expanding range edge (Dudaniec et al., 2018). A key difference between range shifting and invasive species is the prior existence of shared distributional boundaries (Urban et al., 2012), and populations which undergo range shifts are those which already exist at the cold edge of their distributions, necessitating adaptation to colder environments compared to core populations. This may mean that despite the species’ broad distributional range, local populations have become increasingly cold adapted as they approach their expanding edge, allowing *Ischnura* greater competitive success temperatures more reflective of current field conditions. It is somewhat surprising that *I. elegans* did not perform better in interspecific competition under warmer than colder conditions, given that previous work shows that growth rates of *I. elegans* larvae from these populations (but reared in isolation) are worse than in their native competitors at 15 degrees, but not at 20 degrees (Lancaster et al., 2017). It may be that direct competitive effects reverse this trend in lone growth rates, or that more replication is needed to flesh out these interactive effects. Additionally, the lack of any temperature effects on adult survival (Fig. 5) further indicates that local *Ischnura* populations are competitive within the typical thermal range experienced in high latitude environments, given that adults were subject only to ambient thermal variations rather than experimentally manipulated temperatures.

Biotic Resistance on Resident Success

*Lestes sponsa* were predicted to perform more favourably in more biologically complex competitive treatments, with biotic resistance decreasing the competitive impact experienced from any one species, increasing fitness. However, *Lestes* larvae show no impacts of competitive treatment on any demographic parameters (Fig. 6). As discussed above, *Lestes* larvae are behaviourally quite different from both *Ischnura* and *Enallagma* larvae, with their greater activity likely leading to increased foraging success (Brodin, 2009), which may have allowed them to either avoid or outcompete these other species. As the dominant larval competitor, they would be little affected by changes in species composition within the community, reflecting these results.

As adults, however, *Lestes sponsa* are much more sensitive to the competitive environment, with adult survival depending strongly on competitive treatment (Fig. 7). *Lestes* survival in intraspecific conditions remains consistently high, suggesting that competition for mating opportunity within *Lestes* populations is not so intense as to impact survival. When competing interspecifically with a second species, however, be it a species with which *Lestes* has experienced long-term coexistence (*Enallagma cyathigerum*) or novel a competitor from a recent range shift (*Ischnura elegans*), *Lestes* survival rapidly declines (Fig. 7). This suggests that adult *Lestes sponsa*, unlike their larvae, are not strong competitors, either for food or habitat space, and are poorly equipped to survive the harassment of other species. This coincides with the findings of Fitt and Lancaster (2017), who demonstrate thermal displacement of *Lestes* when in competition with *Ischnura elegans*. Despite their negative impacts on *Lestes* survival, *Ischnura* do not appear to contribute directly to the amount of mating harassment *Lestes* experience, with *Ischnura* males never attempting to copulate with *Lestes* females (Fig. S1). However, the increased amount of direct mating harassment experienced by *Lestes* females in treatments with *Enallagma* present (Fig. S1) reflects the known territoriality of *Enallagma* species (Moody, 2009). *Enallagma* males harass not only female damselflies, but also males of both their own and other species. The greater probability of male-male interaction, given the male biased sex ratios present in both this experiment and natural populations (Stoks, 2001), perhaps also explains the result of greater male mortality seen across species in our experiment (Fig. 7), given the energetic costs associated with such harassment (Gosden and Svensson, 2009). It is in both adult survival and harassment, however, where we find evidence of biotic resistance conferring a competitive advantage to *Lestes sponsa*. Despite the strong competitive impacts of both these species, *Lestes* survival is much improved in the multispecies treatments compared to in competition with the range shifter *I. elegans* alone (Fig. 7). Interestingly, the presence of the range shifter also improves adult survival over the native-native interspecific competition scenario, as the presence of *I. elegans* eliminates direct mating harassment from *Enallagma* (Fig. S1). This suggests that the presence of multiple competitors may indeed relieve some of the competitive strength experienced by *Lestes* in these more complex treatments, supporting biotic resistance as a beneficial mechanism for the survival and success of native species experiencing competitive stress from either a native competitor or a novel range shifter.


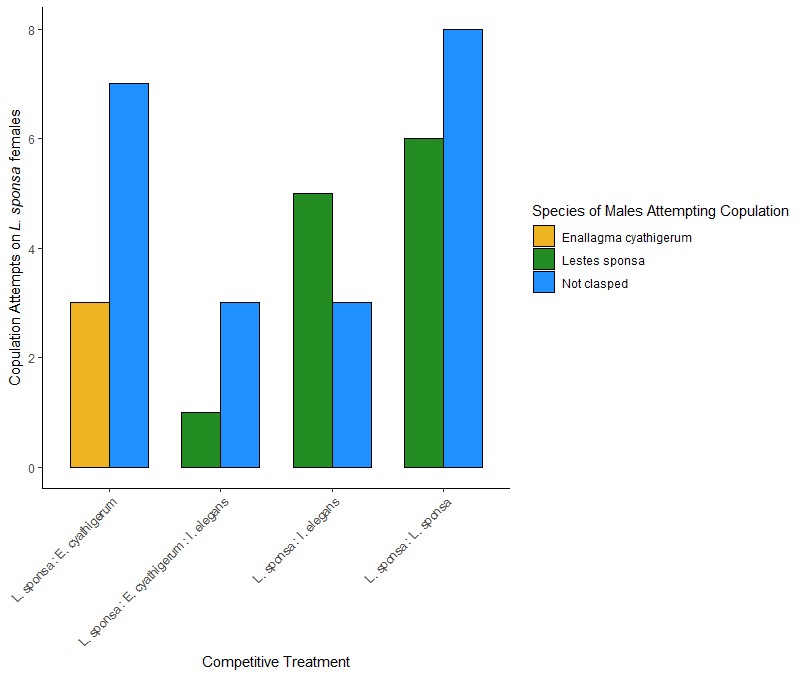


Figure S1. Raw data for the attempted copulations of male damselflies per female *Lestes* *sponsa*. Mating success/attempt was denoted by the presence of fluorescent powder on the female *Lestes’s* genitals, as opposed to the thorax (which was used to measure mating harassment), with the colour of powder revealing the species that attempted copulation. Given that the response variable regards exclusively female *Lestes*, copulation with male *Lestes* is considered as a success (green), compared to mating attempts with *Enallagma* (yellow) or indeed a lack of any mating attempt (blue). Unfortunately, sample size proved too small to allow the fitting of complex generalized linear mixed models to this data. Nonetheless, the raw data presents a trend of decreasing mating success (with fewer *L. sponsa* males attempting copulation with *L. sponsa* females/transferring powder to female genitalia) with both increasing community complexity, and notably, increasing presence of *E. cyathigerum,* suggesting that the presence of *E. cyathigerum* males impinges strongly on the mating success of *Lestes*. *Ischnura* males never attempted to copulate with *Lestes* females.

Temperature Effects on Resident Success

*Lestes sponsa* were additionally predicted to experience reduced fitness in warmer temperatures, given their exclusivity to higher latitude environments and the anticipated advantage such conditions would create for their range shifting competitors. Temperature effects are indeed present in the best fit models for each life history parameter of *Lestes* larvae; however, the lack of any strong negative impact of higher temperatures suggests that *Lestes* larvae are able to remain competitive across a range of thermal conditions to a greater extent than anticipated. Their ability to maintain survival and growth rates in warmer conditions, despite being a high latitude species, is perhaps explained by their comparatively later emergence times, with *Lestes* larvae being active in the height of summer and only emerging as adults in July/August (Śniegula and Johansson, 2010). Such phenological separation between species is likely an adaptation to reduce the strength of competition experienced by adult *Lestes* (Harabiš et al., 2012), further supporting the idea of *Lestes* adults being weak interspecific competitors. However, this later emergence may have additionally facilitated the adaptation of *Lestes* larvae to the warmer water temperatures typically present later in the season, allowing them to remain competitively viable against novel range shifting competitors even in increasing thermal regimes.

As adults however, temperature strongly impacts *Lestes* survival, a result perhaps explained again by the relationship between ambient temperature and energy availability (Nilsson-Örtman et al., 2014). At colder temperatures individuals are less active and less aggressive, allowing *Lestes* to avoid much of the negative impact of interspecific competition. As temperatures increase, however, competitor energy also increases, with competitors becoming more aggressive and territorial, impacting *Lestes* survival (Fig. 7). However, in the multispecies treatment, while increasing temperatures still results in competitors becoming more active and aggressive, the presence of additional species allows *Lestes* to avoid competition and harassment, improving survival. This synthesis is also supported by the findings of Fitt and Lancaster (2017), who find increasing densities of *Lestes sponsa* in the field as temperature increases in the absence of interspecific competition, but decreasing *Lestes* densities with temperature when *Ischnura* are present. Together, these results support the idea of temperature mediating competitive impacts, and of interactions between increasing temperatures and biotic resistance conferring fitness advantages to a resident species.

References

Brodin, T., 2009. Behavioral syndrome over the boundaries of life—carryovers from larvae to adult damselfly. Behav. Ecol. 20, 30–37. https://doi.org/10.1093/beheco/arn111

Brodin, T., Johansson, F., 2004. Conflicting selection pressures on the growth/predation-risk trade-off in a damselfly. Ecology 85, 2927–2932. https://doi.org/10.1890/03-3120

Dudaniec, R.Y., Yong, C.J., Lancaster, L.T., Svensson, E.I., Hansson, B., 2018. Signatures of local adaptation along environmental gradients in a range‐expanding damselfly ( *Ischnura elegans* ). Mol. Ecol. 27, 2576–2593. https://doi.org/10.1111/mec.14709

Duriez, O., Fritz, H., Binet, F., Tremblay, Y., Ferrand, Y., 2005. Individual activity rates in wintering Eurasian woodcocks: starvation versus predation risk trade-off? Anim. Behav. 69, 39–49. https://doi.org/10.1016/j.anbehav.2004.04.009

Gosden, T.P., Svensson, E.I., 2009. Density‐Dependent Male Mating Harassment, Female Resistance, and Male Mimicry. Am. Nat. 173, 709–721. https://doi.org/10.1086/598491

Harabiš, F., Dolný, A., Šipoš, J., 2012. Enigmatic adult overwintering in damselflies: coexistence as weaker intraguild competitors due to niche separation in time. Popul. Ecol. 54, 549–556. https://doi.org/10.1007/s10144-012-0331-8

Lancaster, L.T., Dudaniec, R.Y., Hansson, B., Svensson, E.I., 2015. Latitudinal shift in thermal niche breadth results from thermal release during a climate‐mediated range expansion. J. Biogeogr. 42, 1953–1963. https://doi.org/10.1111/jbi.12553

Lancaster, L.T., Morrison, G., Fitt, R.N., 2017. Life history trade-offs, the intensity of competition, and coexistence in novel and evolving communities under climate change. Philos. Trans. R. Soc. B Biol. Sci. 372, 20160046. https://doi.org/10.1098/rstb.2016.0046

Levine, J.M., Adler, P.B., Yelenik, S.G., 2004. A meta‐analysis of biotic resistance to exotic plant invasions. Ecol. Lett. 7, 975–989. https://doi.org/10.1111/j.1461-0248.2004.00657.x

Martin, T.E., Scott, J., Menge, C., 2000. Nest predation increases with parental activity: separating nest site and parental activity effects. Proc. R. Soc. Lond. B Biol. Sci. 267, 2287–2293. https://doi.org/10.1098/rspb.2000.1281

McPeek, M.A., 2004. The Growth/Predation Risk Trade‐Off: So What Is the Mechanism? Am. Nat. 163, E88–E111. https://doi.org/10.1086/382755

Nilsson-Örtman, V., Stoks, R., Johansson, F., 2014. Competitive interactions modify the temperature dependence of damselfly growth rates. Ecology 95, 1394–1406. https://doi.org/10.1890/13-0875.1

Schaffner, A.K., Anholt, B.R., 1998. Influence of Predator Presence and Prey Density on Behaviour and Growth of Damselfly Larvae (Ischnura elegans) (Odonata: Zygoptera). J. Insect Behav. 11, 793–809. https://doi.org/10.1023/A:1020803925186

Śniegula, S., Johansson, F., 2010. Photoperiod affects compensating developmental rate across latitudes in the damselfly *Lestes sponsa*. Ecol. Entomol. 35, 149–157. https://doi.org/10.1111/j.1365-2311.2009.01164.x

Start, D., Kirk, D., Shea, D., Gilbert, B., 2017. Cannibalism by damselflies increases with rising temperature. Biol. Lett. 13, 20170175. https://doi.org/10.1098/rsbl.2017.0175

Stoks, R., 2001. What causes male‐biased sex ratios in mature damselfly populations? Ecol. Entomol. 26, 188–197. https://doi.org/10.1046/j.1365-2311.2001.00303.x

Stoks, R., 1999. Autotomy shapes the trade-off between seeking cover and foraging in larval damselflies. Behav. Ecol. Sociobiol. 47, 70–75. https://doi.org/10.1007/s002650050651

Strobbe, F., McPeek, M.A., De Block, M., Stoks, R., 2011. Fish predation selects for reduced foraging activity. Behav. Ecol. Sociobiol. 65, 241–247. https://doi.org/10.1007/s00265-010-1032-y

Svensson, E.I., 2012. Non-ecological speciation, niche conservatism and thermal adaptation: how are they connected? Org. Divers. Evol. 12, 229–240. https://doi.org/10.1007/s13127-012-0082-6

Urban, M.C., De Meester, L., Vellend, M., Stoks, R., Vanoverbeke, J., 2012. A crucial step toward realism: responses to climate change from an evolving metacommunity perspective. Evol. Appl. 5, 154–167. https://doi.org/10.1111/j.1752-4571.2011.00208.x

Willink, B., Ware, J.L., Svensson, E.I., 2024. Tropical Origin, Global Diversification, and Dispersal in the Pond Damselflies (Coenagrionoidea) Revealed by a New Molecular Phylogeny. Syst. Biol. syae004. https://doi.org/10.1093/sysbio/syae004

Wooster, D., Sih, A., 1995. A Review of the Drift and Activity Responses of Stream Prey to Predator Presence. Oikos 73, 3. https://doi.org/10.2307/3545718
